# The Effects of the Crohn's Disease Exclusion Diet (CDED) Alone Versus CDED Plus Partial Enteral Nutrition (PEN) on Gut Microbiome Composition in Pediatric CD Patients

**DOI:** 10.1002/mbo3.70099

**Published:** 2025-10-24

**Authors:** Pejman Rohani, Abtin Ansari, Mohsen Shaygantabar, Azita Hekmatdoost, Mohammad Hassan Sohouli

**Affiliations:** ^1^ Pediatric Gastroenterology and Hepatology Research Center, Pediatrics Centre of Excellence, Children's Medical Center Tehran University of Medical Sciences Tehran Iran; ^2^ School of Medicine Tehran University of Medical Science Tehran Iran; ^3^ Department of Clinical Nutrition and Dietetics, Faculty of Nutrition Sciences and Food Technology, National Nutrition and Food Technology Research Institute Shahid Beheshti University of Medical Science Tehran Iran

**Keywords:** Crohn's disease, exclusion diet, *Faecalibacterium prausnitzii*, gut microbiome, partial enteral nutrition, pediatric

## Abstract

Crohn's disease (CD) is a chronic inflammatory bowel condition characterized by relapsing inflammation and microbial dysbiosis. Diet‐based therapies have emerged as promising adjuncts in pediatric CD management. To our knowledge, this is the first randomized trial to directly compare Crohn's Disease Exclusion Diet (CDED) with and without Partial Enteral Nutrition (PEN) in pediatric Crohn's patients from a microbiome perspective, highlighting the potential clinical relevance of dietary strategies that are more feasible than exclusive enteral nutrition. In this randomized controlled trial, 60 children with mild‐to‐moderate CD were assigned to either a CDED‐only group or a CDED + PEN group. Gut microbiota composition was assessed using quantitative PCR before and after the 8‐week intervention. After 8 weeks, both groups exhibited significant increases in *Faecalibacterium prausnitzii*, with the CDED + PEN group demonstrating a significantly greater increase compared to the CDED group alone (+8.94 vs. +5.00 log CFU, *p* < 0.001). In the CDED + PEN group, levels of *Bifidobacterium* and *Lactobacillus* also rose significantly, whereas in the CDED‐only group, *Bifidobacterium* slightly decreased and *Lactobacillus* showed only a modest increase. Moreover, a significant reductions in *Escherichia coli* and *Fusobacterium* were observed in the CDED + PEN group compared to CDED alone (*E. coli*, –1.44 log CFU, *p* < 0.001; *Fusobacterium*, –1.08 log CFU, *p* < 0.001). Changes in *Clostridium leptum* and *Ruminococcus* were minimal and not statistically significant between or within groups. These findings suggest a synergistic effect of CDED when combined with PEN in modulating the gut microbiome toward a more anti‐inflammatory profile. These findings suggest that CDED + PEN may enhance microbiome modulation compared to CDED alone; however, given the modest sample size and 8‐week follow‐up, these results should be interpreted cautiously.

## Graphical abstract

“In this randomized trial in pediatric Crohn's disease, the Crohn's Disease Exclusion Diet combined with partial enteral nutrition more effectively enhanced beneficial gut microbes (Faecalibacterium, Bifidobacterium, Lactobacillus) and reduced pro‐inflammatory taxa (*E. coli*, Fusobacterium) than diet alone, highlighting the synergistic microbiome benefits of this dietary approach.

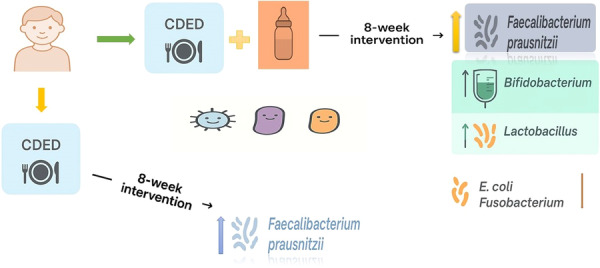

## 1 Introduction

Crohn disease (CD) is an inflammatory bowel disease (IBD) which has mostly insidious trend in contrast of ulcerative colitis (UC). Together, they represent the two main subtypes of IBD. The incidence of CD has been rising worldwide in recent decades, including in both Western and Asian pediatric populations (Malaty et al. 2010; Shen et al. 2011; Kim and Ferry 2004; Cosnes et al. 2011). CD is considered an immune‐mediated disease which can make inflammation in entire thickness of tissue through gastrointestinal tract (Ranasinghe et al. 2025). CD can be manifested as periods of relapse and remission, and can also manifest itself with numerous digestive symptoms, including diarrhea and abdominal pain. Fistulas in the perianal area and strictures in the digestive tract can be complications of this disease. In children, this disease can cause complications, including delayed growth and malnutrition, and have a negative impact on the mental health, social behaviors, and quality of life (Kim and Ferry 2004; Diefenbach. 2006; Mackner and Crandall 2007).

Although, etiology of CD is unclear, there is a strong hypothesis about development of CD, which mention that it would be emerged under interaction between genes, environmental factors, and immune system response (Kaser et al. 2010; Lees et al. 2011). There are various factors, including smoking, appendectomy, diet, drugs, and microbes, and enteric flora are associated with the development and flare of CD (Danese et al. 2004). Microbial dysbiosis, characterized by reduced diversity and altered abundances of commensal taxa, has been consistently reported in pediatric CD and may contribute to relapse and remission dynamics (Round and Mazmanian 2009; Sartor 2008; González‐Torres et al. 2022; Manichanh et al. 2012). Immune responses to dietary and environmental antigens may further contribute to intestinal inflammation in children with CD, highlighting the overlap between food antigens, allergens, and mucosal immune activation (Zhou et al. 2022).

Among these factors, the Crohn's Disease Exclusion Diet (CDED) and exclusive enteral nutrition (EEN) have demonstrated efficacy in inducing remission and modulating the gut microbiota. However, the strict adherence required for EEN often limits its long‐term feasibility in routine clinical practice (Heuschkel et al. 2000; van Rheenen et al. 2020). To address this limitation, Partial Enteral Nutrition (PEN), when combined with specific exclusion diets like CDED, has been proposed as a more sustainable yet still effective dietary approach. While emerging data suggest that this combination may be clinically effective, there is a notable lack of mechanistic insight into how these diets, individually and in combination, influence the gut microbiome in pediatric Crohn's disease. Most studies to date have focused on clinical outcomes or have assessed microbiome changes in response to EEN alone, often neglecting real‐world, more feasible dietary patterns like CDED + PEN (González‐Torres et al. 2022). Furthermore, the existing literature rarely includes direct, comparative analyses of the gut microbial shifts associated with exclusion diets with versus without adjunctive PEN. The dynamic interplay between diet, microbiota composition, and inflammation is complex, and it remains unclear whether the addition of PEN confers additional microbiological or immunological benefits beyond those provided by the exclusion diet alone (Sigall Boneh et al. 2024; Rajendran and Kumar 2011; Sigall‐Boneh et al. 2014).

Based on these gaps, we hypothesized that the combination of CDED with partial enteral nutrition would induce more favorable shifts in the gut microbiome compared to CDED alone, providing mechanistic insight into the clinical benefits observed in pediatric Crohn's disease.

## 2 Methods

### 2.1 Trial Design

This was a randomized controlled parallel‐group trial with 1:1 allocation ratio which was conducted in children medical center hospital, Tehran, Iran.

We aimed to assess the impact of these two following interventions on gastrointestinal (GI) microbiome composition in children with age between 4 and 18 years with mild to moderate CD, confirmed through clinical, radiological, endoscopic, and histopathological evaluations. Interventions are CDED plus PEN in one group and only CDED in another group of study.

### 2.2 Eligibility Criteria for Participants

All pediatric patients visiting the centre's clinic between the ages of 4 and 18 who have with mild to moderate CD can enter the study, provided they have pediatric Crohn disease activity index of ≥ 10 and ≤ 40. Participants showed evidence of active inflammation at referral, indicated by C‐reactive protein (CRP) levels above 5 g/L, erythrocyte sedimentation rate (ESR) exceeding 20 mm/hour, or fecal calprotectin levels greater than 200 µg/g. Exclusion criteria (Appendix S1) encompassed patients with stenosis, small bowel obstruction, prior intestinal resection, or a history of biological treatment. Additionally, individuals currently using steroids, those who started immunomodulatory therapy within the previous 8 weeks, or those with a pediatric Crohn's disease activity index (PCDAI) score below 10 or above 40 were excluded. Maintenance therapy with fixed doses of immunomodulators, such as thiopurines or methotrexate, was allowed at baseline or during the 3‐week follow‐up, consistent with standard clinical practice for nutritional interventions in pediatric patients. These agents are considered to have an effect only after 8 weeks and are not believed to impact the disease during this period. All patients who were under administration of drugs like steroids or methotrexate, 5‐ASA, antibiotics or any other drugs which can induces remission phase, are excluded from the study. More details about inclusion/exclusion criteria have been shown in Appendix S1.

### 2.3 Interventions

Patients were randomly assigned to CDED + PEN or CDED interventions. All participants underwent evaluations at baseline and at the 8‐week follow‐up. Patients also received a detailed list of permitted and prohibited foods (Appendix S2), accompanied by thorough guidance from a nutritionist. CDED is a established intervention designed to decrease exposure to dietary elements that may exacerbate inflammation, disrupt the gut microbiota or the intestinal barrier. The diet focus on eliminating specific food groups which considered as pro‐inflammatory or detrimental to gut health. In the first intervention group, with the exclusion diet, one of two palatable formulas, each providing 1 Kcal/mL and available in Iran—Resource Junior (Nestle, Vevey, Switzerland) or Pediasure (Abbott B.V., Hoofddorp, Netherlands)—was administered according to the patient's option, irrespective of age. The calculated volume of the formula was established based on the current weight to guarantee that 50% of energy was sourced from the formula, with a ceiling of 1250 kcal/d. Only certain spices and herbs were allowed, whilst all other condiments and sauces were disregarded. Specifically, substances containing gluten, dairy products, gluten‐free baked goods and breads, animal‐derived fats, processed meats, items with emulsifiers, iron supplements, margarine, butter, vinegar, mayonnaise, ketchup, soft drinks, desserts, juices, fried or oily foods, and canned goods were prohibited. The diet has a minimum of 18– 20 g of fiber daily. The elimination diet (Appendix S2) comprises five essential items ingested daily: chicken breast, two eggs, two bananas, one apple, and two medium potatoes. These potatoes must be cooked using techniques such as baking, boiling, or broiling, and then chilled before to eating, since this alters the starch composition. An essential component of the diet is the exclusion of items not included in the authorized or requisite list. The barred articles had more significance than those authorized, and replacements were prohibited. Patients who abstain from meat may ingest more formula. Calcium supplements were given to individuals who refused formula.

### 2.4 Outcome of Interest

The primary outcome was the change in gut microbiota composition (relative abundance of pre‐specified bacterial taxa) from baseline to week 8. Secondary outcomes included clinical disease activity indices (PCDAI, CRP, ESR, and fecal calprotectin). In the initial study on which the protocol was designed, the primary objective was to investigate the effectiveness of these two interventions (the protocol has been registered in protocol ID gov (NCT06353633, with a registration date of 2024‐02‐04). In this study, we specifically investigated the effect of these two interventions on the composition of gut microbes obtained from the stool of Crohn's patients.

### 2.5 Genomic DNA Extraction, and Analysis of the Microbiome Composition

Fecal specimens, weighing 10 g, were collected from participants and preserved in opaque containers designed for stool storage. Immediately post‐collection, the samples were flash‐frozen at −80°C and maintained under these conditions until subsequent analysis. Bacterial DNA was isolated from the fecal material utilizing the FavorPrepTM Stool DNA Isolation Mini Kit (Favorgen Biotech Corp, Pingtung, Taiwan), adhering strictly to the protocol provided by the manufacturer. Quantitative real‐time polymerase chain reaction (qPCR) was employed to detect and quantify the bacterial populations within the samples. Genus‐specific primers were applied, with the 16S RNA gene of *Escherichia coli* serving as the reference standard. The abundance of each bacterial taxon was determined by comparing the amplification curves to a standard curve, derived from serial dilutions of the target bacteria, using the same qPCR platform. According to previous studies, *Faecalibacterium prausnitzii*, *Bacteroides, Ruminococcus, Bifidobacterium, Lactobacillus, Clostridium leptum, Escherichia coli*, and *Fusobacterium* were quantified in our study.

### 2.6 Sample Size Calculation

The sample size was calculated based on PCDAI changes, which was the primary clinical outcome of the parent trial, in agreement with the study of Sigall‐Boneh et al (Sigall‐Boneh et al. 2014). This limitation is acknowledged To assess the impact of an exclusion diet in combination with PEN on CD, a minimum difference of 10 points in the mean PCDAI was deemed significant between the intervention group (receiving both treatment) and the control group (receiving only the exclusion diet). The appropriate sample size per group was calculated with the standard formula, with a two‐sided 5% significance level and a power of 80%, yielding 25 participants in each group. To account for an anticipated dropout rate of 20%, the final sample size was adjusted to include 30 participants per group.

### 2.7 Randomization

Patients were assigned to groups in a 1:1 ratio through block randomization, using blocks of six to ensure balance. The randomization sequence was created in advance with Microsoft Excel (Microsoft, Redmond, WA) to promote consistency and reduce bias. To maintain allocation concealment, randomization codes were sealed in sequentially numbered, opaque envelopes, which kept group assignments hidden until the moment of allocation. The envelopes were opened only after obtaining both informed consent from the patients and their assent to participate in the randomization process, in accordance with ethical guidelines. To further safeguard allocation concealment, the randomization codes were inaccessible to the physicians involved in patient enrollment, thereby mitigating the risk of selection bias. This robust methodology ensured strict adherence to randomization protocols and enhanced the internal validity of the trial.

### 2.8 Statistical Analysis

The endpoint of this study was changes in gut microbiome after 8 weeks of two determined interventions. The demographic and baseline characteristics of participants were shown in Table 1 which quantitative variables are reported by mean with standard deviation and qualitative variables are reported by percentage. To assess quantitative variables between two groups, we employed an independent sample *t*‐test, while categorical variables were analyzed using the chi‐square test. Between‐group differences in bacterial taxa were assessed using ANCOVA, adjusted for baseline values, fiber intake, and concomitant medication use. To account for multiple comparisons across bacterial taxa, [FDR correction at q < 0.05/Bonferroni correction] was applied. Where results remained significant after correction, they are reported accordingly. Analyses were performed using SPSS version 26. We aimed to assess the effect of these two interventions on composition of gut microbiome and comparison between these two interventions.

**Table 1:** Comparison of baseline characteristics of the participants.

Variable		CDED + PEN (*n* = 30)	CDED (*n* = 30)	*p* value
Age (year)		11.80 ± 3.66	11.93 ± 3.32	0.88
Gender	Male	17 (56.7%)	12 (41.4%)	0.30
	Female	13 (43.3%)	17 (58.6%)	
BMI (kg/m^2^)		18.50 ± 3.58	17.01 ± 3.23	0.11
Weight (kg)		39.68 ± 14.45	32.17 ± 14.70	0.06
Hemoglobin (g/dL)		11.00 ± 2.13	10.98 ± 2.15	0.97
Disease activity (based on PCDAI)		24.40 ± 9.12	23.89 ± 7.51	0.81
CRP (mg/L)		34.33 ± 34.38	30.68 ± 24.00	0.64
ESR (mm/h)		40.89 ± 21.47	37.68 ± 14.72	0.51
Alb (g/L)		3.66 ± 0.68	3.86 ± 0.76	0.32
Calprotectin (µg/g)		1503.82 ± 1373.35	1600.67 ± 941.79	0.75
Family history of IBD *n* (%)		4 (13.8%)	8 (27.6%)	0.33
Comorbidity *n* (%)		8 (28.6%)	11 (40.7%)	0.40
Azathioprine use *n* (%)		12 (40%)	11 (37.9%)	1.00
MTX use *n* (%)		6 (20%)	7 (24.1%)	0.76

*Note:* Data represented as mean ± SD or *n* (%).

Independent sample *t*‐test for quantitative variables, chi‐square test for categorical variables.

Abbreviations: Alb, albumin; BMI, body mass index; CRP, C‐reactive protein; ESR, erythrocyte sedimentation rate; MTX, methotrexate; PCDAI, pediatric Crohn's disease activity index.

## 3 Results

### 3.1 Baseline Characteristics

**Figure 1:**
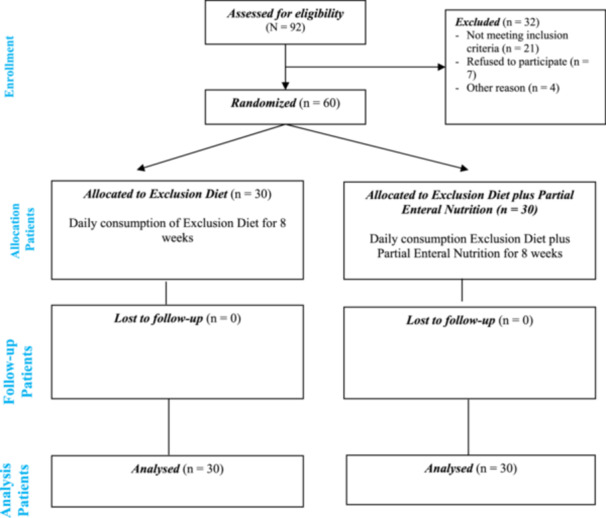
Consort flow diagram for the trial.

Sixty patients were evenly randomized into two cohorts (30 assigned to CDED + PEN and 30 to CDED) and included in the statistical evaluation (Figure 1). The baseline characteristics of the participants were comparable between the two groups (CDED + PEN vs. CDED) with no statistically significant differences observed (Table 1). The mean age of participants was 11.80 ± 3.66 years in the CDED + PEN group and 11.93 ± 3.32 years in the CDED group (*p* = 0.88). Gender distribution was similar (*p* = 0.30), with 56.7% males in the CDED + PEN group and 41.4% in the CDED group. Anthropometric parameters including BMI (18.50 ± 3.58 vs. 17.01 ± 3.23 kg/m², *p* = 0.11), and weight (39.68 ± 14.45 vs. 32.17 ± 14.70 kg, *p* = 0.06) did not differ significantly.

Laboratory indices including hemoglobin, CRP, ESR, albumin, as well as disease activity (PCDAI), family history of IBD, comorbidities, and use of azathioprine or methotrexate were all statistically comparable between groups (all *p* > 0.05).

### 3.2 Gut Microbiome Composition

As shown in Table 2, the intervention had significant impacts on gut microbiome composition within and between groups. At week 8, distinct changes in gut microbiota profiles were observed between groups. The both groups (CDED + PEN and CDED alone group) demonstrated a significant increase in *Faecalibacterium prausnitzii (F. prausnitzii)* (mean changes: +8.94 log CFU and + 5.00 log CFU, respectively; *p* < 0.001), resulting in a highly significant between‐group difference (*p* < 0.001). Similarly, beneficial bacteria including *Bifidobacterium and Lactobacillus* increased significantly in the CDED + PEN group (mean changes: +1.94 and +1.71 log CFU, respectively; *p* < 0.001), whereas the CDED group showed a nonsignificant reduction in *Bifidobacterium* (–0.39, *p* = 0.102) and a modest increase in *Lactobacillu*s (+0.44, *p* = 0.039). Also, significant changes were observed in *Bacteroides* in both groups after 8 weeks, as it decreased in the CDED + PEN group and increased in the CDED group, which ultimately indicated significant changes between the two groups after 8 weeks of intervention. Notably, *Escherichia coli (E. coli)* and *Fusobacterium* levels significantly decreased in the CDED + PEN group (–1.44 and –1.08 log CFU, respectively; both *p* < 0.001), but remained unchanged in the CDED group, with significant between‐group differences for both (*p* < 0.001 and *p* = 0.001, respectively).

**Table 2:** Gut microbiome composition before and after the intervention in CDED + PEN and CDED groups.

Variable	Groups	Before	Changes from baseline at week 8	*p* value^a^
*Faecalibacterium prausnitzii*. Log CFU	*CDED* + *PEN*	6.05 ± 1.06	8.94 ± 1.43	< 0.001
	*CDED*	6.75 ± 1.01	5.00 ± 1.40	< 0.001
	*P‐value* ^*2*^	0.010	< 0.001	
Bacteroides. Log CFU	*CDED* + *PEN*	10.22 ± 0.87	−0.88 ± 1.09	< 0.001
	*CDED*	10.17 ± 1.08	1.22 ± 1.47	< 0.001
	*P‐value* ^*2*^	0.820	< 0.001	
Bifidobacterium. Log CFU	*CDED* + *PEN*	5.31 ± 0.75	1.94 ± 1.55	< 0.001
	*CDED*	5.20 ± 0.96	−0.39 ± 1.26	0.102
	*P‐value* ^*2*^	0.621	< 0.001	
Lactobacillus. Log CFU	*CDED* + *PEN*	3.52 ± 0.89	1.71 ± 1.43	< 0.001
	*CDED*	3.68 ± 1.29	0.44 ± 1.11	0.039
	*P‐value* ^*2*^	0.561	< 0.001	
*Escherichia coli*. Log CFU	*CDED* + *PEN*	5.27 ± 1.19	−1.44 ± 1.52	< 0.001
	*CDED*	5.01 ± 1.20	0.17 ± 1.18	0.439
	*P‐value* ^*2*^	0.403	< 0.001	
*Clostridium leptum*. Log CFU	*CDED* + *PEN*	2.87 ± 0.91	0.24 ± 1.00	0.161
	*CDED*	2.75 ± 0.99	0.20 ± 0.89	0.222
	*P‐value* ^*2*^	0.638	0.881	
*Ruminococcus*. Log CFU	*CDED* + *PEN*	8.30 ± 0.99	0.51 ± 0.96	0.003
	*CDED*	8.29 ± 0.94	0.25 ± 0.89	0.130
	*P‐value* ^*2*^	0.978	0.280	
*Fusobacterium*. Log CFU	*CDED* + *PEN*	4.15 ± 1.04	−1.08 ± 1.18	< 0.001
	*CDED*	4.22 ± 1.14	0.05 ± 1.40	0.844
	*P‐value* ^*2*^	0.807	0.001	

*Note:* Data repressented as mean ± SD.

^a^ within group comparison at first follow up (Baseline and week 8), paired *t*‐test, 2: *p*‐value changes for between‐group differences using analyses of covariance, considering baseline values, fiber intake, and drug use as covariate.

On the other hand, changes in *Clostridium leptum* and *Ruminococcus* were minimal and not statistically significant in either group or between groups. For instance, *Clostridium leptum* increased slightly in both groups (mean change +0.24 in CDED + PEN and +0.20 in CDED), but neither change was significant (*p* = 0.161 and 0.222), and no between‐group difference was found (*p* = 0.881). Similarly, *Ruminococcus* showed a minor increase in the CDED + PEN group (+0.51, *p* = 0.003), but this was not statistically different from the change observed in the CDED group (+0.25, *p* = 0.130; between‐group *p* = 0.280).

## 4 Discussion

In this study, the intervention significantly altered gut microbiome composition, with distinct effects observed between the CDED + PEN and CDED groups by week 8. The CDED + PEN group exhibited a marked increase in beneficial bacteria, including a significant rise in *Faecalibacterium prausnitzii* (*p* < 0.001), *Bifidobacterium* (*p* < 0.001), and *Lactobacillus* (*p* < 0.001), alongside significant reductions in *Bacteroides, Escherichia coli* (*E. coli*) and *Fusobacterium* (both *p* < 0.001). In contrast, the CDED group showed a significant decrease in *F. prausnitzii* (*p* < 0.001), a nonsignificant reduction in *Bifidobacterium* (*p* = 0.102), and a modest increase in *Bacteroides* and *Lactobacillus* (*p* = 0.039), with no notable changes in *E. coli* or *Fusobacterium*. Finally, significant changes were reported between the two intervention groups for all bacteria studied except *Clostridium leptum* and *Ruminococcus*. These findings suggest that the combination of CDED and PEN not only enhances the abundance of key beneficial microbial populations but also significantly reduces pro‐inflammatory and potentially pathogenic taxa more effectively than CDED alone.

This study was conducted as part of a study investigating the effect of these two intervention related to diets on inducing remission in children with Crohn's disease. Given the importance and need for further explanation and elaboration in the field of the relationship with the intestinal microbiome, it was decided to examine and analyze it in a separate study.

According to the findings of this study, people who received CDED + PEN and CDED alone had a significant increase in the population of bacteria *F. prausnitzii*. However, the incremental changes in the CDED + PEN group were significantly greater than those in the CDED group. The observed increase in F. prausnitzii may contribute to enhanced butyrate synthesis, a short‐chain fatty acid with mucosal protective and anti‐inflammatory effects. Also, the beneficial expansion of F. prausnitzii observed here may be mechanistically linked to butyrate production, which modulates host epigenetic and metabolic pathways, as recently demonstrated in cardiovascular pathology (Wang et al. 2025). PEN may provide substrates that favor expansion of beneficial taxa; however, we did not directly measure metabolites such as butyrate or functional immune responses (Gisbert and Chaparro 2019; Sokol et al. 2008), and therefore these mechanisms remain speculative. Furthermore, the results are consistent with previous studies that believe this bacterium should be considered a recognized taxa which is assumed to have beneficial effects in protecting the intestinal barrier and could play a key role in the pathogenesis of IBD (Sokol et al. 2008; Martín et al. 2014). Previous studies have shown that the population of *F. prausnitzii* in the gut decreases during the active phase of CD (Martinez‐Medina et al. 2006; Kang et al. 2010). Another study that examined the intestinal microbiome composition of children with Crohn's disease who were newly diagnosed and did not receive treatment showed that *F. Prausnitzii* was more concentrated in people with the CD than in healthy people, which calls into question the protective role of this bacteria (Hansen et al. 2012). *F. prausnitzii* also exhibits protective effects by secreting metabolites that inhibit NF‐κB secretion and by inducing the secretion of anti‐inflammatory cytokines (Sokol et al. 2008).

The divergent changes observed in *Bacteroides* abundance between the two groups suggest that dietary composition exerts a selective pressure on gut microbiota dynamics. The reduction of *Bacteroides* in the CDED + PEN group, contrasted with its increase in the CDED‐only group, may reflect differences in fiber availability, macronutrient profiles, or enteral formulation content. Prior studies have shown that *Bacteroides* abundance is closely tied to dietary fat and animal protein intake, often increasing in Westernized dietary patterns and decreasing with plant‐rich interventions (Wu et al. 2011; De Filippo et al. 2010). The decline in the CDED + PEN arm may align with findings from partial enteral nutrition protocols that reduce microbial diversity and selectively suppress specific taxa (Hartikainen et al. 2024). Conversely, the CDED alone, being richer in whole‐food components, may have supported *Bacteroides* proliferation. Based on Gerasimidis et al. study, It is worth noted that children who responded to EEN has decreased in Bacteroides species concentration Which could raise the hypothesis that this bacterium plays a role in the initiation or dissemination of intestinal inflammation (Gerasimidis et al. 2014). Given *Bacteroides*' role in mucin degradation and modulation of inflammatory responses, these findings may have implications for therapeutic targeting in Crohn's disease and warrant further mechanistic exploration.

Moreover, *Bifidobacterium* and *Lactobacillus* also increased robustly in the CDED + PEN group than to CDED‐alone group, possibly reflecting the stabilizing effect of PEN on gut pH and mucosal energy supply, which creates a more favorable environment for lactic acid bacteria. These genera are known to enhance epithelial barrier integrity and regulate immune responses via SCFA production and IL‐10 modulation (Bron et al. 2017; O'callaghan and Van Sinderen 2016).

In contrast, levels of *Escherichia coli* and *Fusobacterium*, two taxa strongly associated with mucosal inflammation and microbial dysbiosis in Crohn's disease, declined significantly only in the CDED + PEN group. This may be explained by the restricted availability of nitrogenous and pro‐inflammatory substrates (e.g., animal protein and sulfate) in the combined intervention, which limits growth of these pathobionts. The reduction of *E. coli* and *Fusobacterium*, both implicated in mucosal inflammation and epithelial disruption, suggests a possible mechanistic link between the intervention and improved intestinal barrier function. Prior studies have linked Fusobacterium nucleatum to mucosal invasion and epithelial disruption via E‐cadherin binding and inflammation‐driven adherence (Wu et al. 2022), while *E. coli*, particularly AIEC strains, are known to thrive in inflamed mucosa and utilize nitrate/nitrite produced under oxidative stress (Winter et al. 2013). PEN may reduce such inflammatory niches by promoting epithelial repair and reducing local oxidative damage, thereby preventing expansion of these species. This is consistent with evidence that microbiome alterations interact with oxidative stress and inflammatory pathways, as recently highlighted in multi‐omics analyses of gut–lung axis interactions (Sajid et al. 2025).

The lack of significant change in *Clostridium leptum* and *Ruminococcus* in both groups could be due to their slower growth kinetics and greater dependence on long‐term dietary fiber diversity. These taxa typically flourish under diets rich in resistant starch and complex polysaccharides, which may not have been sufficiently abundant in either intervention phase. Similar temporal dynamics were noted in previous microbiome‐targeted interventions, where *Ruminococcus* populations increased only after ≥ 12 weeks of sustained dietary modulation (Halmos et al. 2015).

Changes in microbiome composition similar to the study conducted do not necessarily mean entering a remission phase in this disease. Or, it cannot be inferred that manipulation of the microbiome composition, if done in this way, can always have a curative role. For example, the results of studies conducted on probiotics such as *Lactobacillus* and *Bifidobacterium* have been very contradictory and practically untrustworthy to this day (Prantera. 2002; Malin et al. 1996). These reveal the complexity and multifaceted nature of this disease, which requires further studies with more advanced analysis models and larger sample sizes, to help uncover the blind spots in the pathogenesis of this disease and provide more appropriate treatments with better patient compliance. One way that enteral nutrition improves gut health is through the beneficial changes it produces in the intestinal microbiota, according to studies by Leach et al. and Lionetti et al. EEN is proposed to contribute to Crohn's disease remission by potentially modulating gut microbiota, including Bacteroides species (Leach et al. 2008). Its presumed therapeutic effect may result from the polymeric liquid formula's low‐residue and prebiotic properties, which could help reduce intestinal inflammation (Lionetti et al. 2005).

Microbial dysbiosis has been recognized as a contributing factor in the development of Crohn's disease. However, the precise mechanisms and pathways through which this imbalance leads to disease onset remain unclear. Additionally, it is yet to be determined whether dysbiosis itself acts as a causative agent of the disease or merely represents a consequence or byproduct of its progression (Manichanh et al. 2012; Kaakoush et al. 2012; Man et al. 2011), But, many studies found that microbial diversity were decreased in CD in comparison to healthy populations (Kaakoush et al. 2015).

Overall, the superior outcomes in the CDED + PEN group may be attributed to synergistic effects of exclusion diet and enteral nutrition: while CDED reduces exposure to pro‐inflammatory and fermentable substrates (e.g., emulsifiers, gluten, lactose), PEN provides consistent caloric intake and bioavailable nutrients that restore epithelial function, immune tolerance, and microbial balance. This synergy appears essential to drive robust increases in beneficial taxa and suppression of dysbiotic strains. These findings are in line with Quince et al. (2015), who also emphasized the complementary effects of nutritional consistency in shaping microbiota trajectories in Crohn's disease patients (Quince et al. 2015). The superior outcomes with CDED + PEN may also be attributed to enhanced mucosal healing, as nutrients are known to modulate macrophage‐driven epithelial repair pathways (Wu et al. 2025).

From a clinical perspective, CDED + PEN may represent a more feasible and sustainable option compared to EEN, particularly in pediatric patients where adherence is a critical barrier. However, the long‐term sustainability and real‐world adherence of such dietary regimens require further study. Furthermore, integration of dietary therapy into digital health platforms could enhance adherence and long‐term monitoring in pediatric CD, consistent with advances in innovation networks and regional digital health system frameworks (Hu et al. 2025). Our findings that dietary modulation impacts microbial taxa resonate with evidence that gut dysbiosis can drive systemic metabolic disturbances through microbiota–host crosstalk, such as in antipsychotic‐induced lipid dysregulation (Zhu et al. 2022). Beyond intestinal inflammation, Crohn's disease often presents with extraintestinal complications, including renal and metabolic disorders (Rosa et al. 2023) (Gras Martínez et al. 2023), underscoring the systemic importance of dietary interventions.

### 4.1 Strengths and Limitations

This study provides robust evidence of the differential impacts of CDED and CDED + PEN on gut microbiome composition focusing on pediatric participants aged 4–18, with significant strengths enhancing its validity. The randomized controlled trial design, with balanced allocation of 30 participants per group, minimized selection bias and ensured comparability between groups, while measuring specific taxa (e.g. *Faecalibacterium prausnitzii, Bifidobacterium*) provides mechanistic insights into the intervention's effects. However, several limitations must be acknowledged. The sample size of 60, while sufficient to detect significant differences in key microbial taxa, may limit the generalizability of results to broader populations, particularly given the wide age range (4–18 years), which could introduce variability in microbiome responses due to developmental differences. Additionally, the 8‐week intervention period, though adequate to observe microbial shifts, may not capture long‐term sustainability or clinical outcomes of the interventions. The study was conducted in a controlled clinical setting, which may not fully reflect real‐world dietary adherence, especially in pediatric populations with varying levels of parental supervision. Furthermore, this study focused on solely microbiome changes without linking to clinical outcomes (e.g. Crohn's disease activity score) which limits the ability to infer therapeutic relevance. The sample size was calculated based on PCDAI changes, which was the primary clinical outcome of the parent trial. The present microbiome analysis represents a secondary endpoint; therefore, the study may not have been fully powered to detect smaller differences in microbial taxa. This limitation is acknowledged. Also, microbiome assessment was limited to qPCR of selected taxa, which, although targeted, does not provide the broader taxonomic or functional insights achievable with 16S rRNA or shotgun metagenomics. Futhermore, we did not perform functional assays (e.g., SCFA quantification, cytokine profiling) to confirm mechanistic interpretations such as butyrate synthesis or NF‐κB modulation. The intervention period was limited to 8 weeks, precluding assessment of long‐term microbiome stability or sustainability of clinical effects. Finally, the modest sample size, although sufficient to detect changes in pre‐specified taxa, limits generalizability. Future studies should employ multi‐omics approaches, longer follow‐up, and larger cohorts to validate the microbiome changes observed and clarify their mechanistic and clinical significance.

## 5 Conclusion

Our study suggests that CDED + PEN leads to more pronounced modulation of commensal gut bacterial composition than CDED alone, particularly with increases in F. prausnitzii and reductions in *E. coli* and Fusobacterium. These findings highlight the potential of dietary interventions to shape the microbiome in pediatric Crohn's disease. However, the study was not designed to establish clinical efficacy, and whether these microbial changes translate into improved patient outcomes remains to be determined. Larger, longer‐term studies incorporating both microbiome and clinical endpoints are needed to validate these observations.

## Author Contributions

**Pejman Rohani:** data curation, writing – original draft, supervision, Writing – review and editing. **Abtin Ansari:** data curation, writing – original draft, Writing – review and editing. **Mohsen Shaygantabar:** data curation, writing – original draft, writing – review and editing. **Azita Hekmatdoost:** conceptualization, methodology, supervision, writing – review and editing. **Mohammad Hassan Sohouli:** conceptualization, methodology, formal analysis, data curation, writing – original draft, writing – review and editing. All authors read and approved the final version of the manuscript.

## Ethics Statement

This study was approved by the research council and ethics committee Tehran University of Medical Sciences, Tehran, Iran (NO: IR.TUMS.CHMC. REC.1403.069). The study has been registered in protocol ID gov (NCT06353633, with a registration date of 2024‐02‐04). Informed written consent was obtained from all participants before the start of the study and they were fully informed about the study objectives and methodology. Moreover, the participants ensured the confidentiality of their information, and they were allowed to leave the study at any time. All participants' information was kept in a personal file which was locked with limited access. The Consolidated Standards of Reporting Trials (CONSORT) guidelines have been followed in this study. Also, written informed consent to participate was obtained from the parents or legal guardians of any participant under the age of 16.

## Consent

The authors have nothing to report.

## Conflicts of Interest

The authors declare no conflicts of interest.

## Supporting information


Appendix S1.

Appendix S2.

## Data Availability

The datasets generated and/or analyzed during the current study are not publicly available due [individual privacy could be compromised] but are available from the corresponding author on reasonable request.
