# Supplementary material for: The Effects of the Crohn's Disease Exclusion Diet (CDED) Alone Versus CDED Plus Partial Enteral Nutrition (PEN) on Gut Microbiome Composition in Pediatric CD Patients
Source: Microbiologyopen. 2025 Oct 24;14(5):e70099. doi: 10.1002/mbo3.70099 (PMC12550864; doi:10.1002/mbo3.70099)
Supplement: Supplementary file 1 — Appendix S1. [file MBO3-14-e70099-s002.pdf]

Appendix S1. Exclusion criteria

1. Patients with evidence of stenosis

2. small bowel obstruction

3. previous intestinal resection

4. PCDAI less than 10 and more than 40

5. Patients who receiving concurrent remission-inducing drugs such as steroids, 5-ASA, methotrexate, or antibiotics, as well as patients receiving induction doses of a biologic, will be excluded. However, patients who will be stable on biologic therapy and had failed or lost response after the first 3 doses of therapy were included in the study as long as no change in biologic dose or schedule will be made.

6. Reluctance to continue the trial

7. Poor adherence to the intervention
