# Supplementary material for: The Effects of the Crohn's Disease Exclusion Diet (CDED) Alone Versus CDED Plus Partial Enteral Nutrition (PEN) on Gut Microbiome Composition in Pediatric CD Patients
Source: Microbiologyopen. 2025 Oct 24;14(5):e70099. doi: 10.1002/mbo3.70099 (PMC12550864; doi:10.1002/mbo3.70099)
Supplement: Supplementary file 2 — Appendix S2. [file MBO3-14-e70099-s001.pdf]

Appendix S2. Exclusion diet instructions

**Mandatory daily foods and quantities**
Fresh chicken breast 150-200 g/d
2 Eggs/ d
2 Bananas/ d
1 Fresh apple/ d
2 Potatoes/ d<br>(Potatoes must be cocked and refrigerated before use)
**Allowed foods daily**
Rice flour
White rice and rice noodles (unlimited)
2 Tomatoes (additional allowed for cooking)
2 Cucumbers (medium size)
2 Avocado halves
Fresh Strawberries
Fresh Melon (1 slice)
1 Carrot
Spinach 1 cup uncooked leaves
Lettuce (3 leaves)
Fresh green herbs (eg, basil, parsley, coriander, rosemary, thyme, mint , dill)
Onion
1 glass freshly squeezed orange juice from fresh oranges (not from cartons or bottles)
Salt, pepper, paprika, cinnamon, cumin, turmeric
Fresh ginger and garlic cloves, lemons and limes
3 tablespoons honey
4 teaspoons sugar
**Foods allowed only once a week**
Fresh lean fish (not deep fried, dietitian guidance required)
**Disallowed foods**
Dairy
Animal fat
Deep-fried or oily foods
Wheat
Emulsifiers
Artificial Sweeteners
Other cuts or parts of chicken
Other sources animal or soy protein
Carrageenans
Maltodextrins(and sucralose)
Sulfite containing foods
Xanthan gum
Packaged, canned or frozen precooked foods, doughs, baked goods
Frozen , canned fruits and vegetables
Oral Iron supplements

Soy or Gluten-free products
Vinegar, soy sauce, ketchup, mayonnaise
Ready to use sauces, syrups, spreads, dressings, margarine, butter
Alcoholic beverages, soft drinks, juices
